# Eculizumab as Additional Rescue Therapy in Myasthenic Crisis

**DOI:** 10.3390/muscles3010005

**Published:** 2024-02-07

**Authors:** Francesco Crescenzo, Mattia Zanoni, Laura Ferigo, Francesca Rossi, Matteo Grecò, Angelica Lupato, Alessandra Danese, Domenico Ajena, Michelangelo Turazzini

**Affiliations:** 1 Neurology Unit, “Mater Salutis” Hospital, AULSS 9 Scaligera, 37045 Verona, Italy; mattia.zanoni@aulss9.veneto.it (M.Z.);; 2 Nephrology and Dialysis Unit, “Mater Salutis” Hospital, AULSS 9 Scaligera, 37045 Verona, Italy

**Keywords:** myasthenia gravis, myasthenic crisis, rescue therapy, eculizumab

## Abstract

Eculizumab is a monoclonal antibody blocking the terminal complement protein C5. As demonstrated in the phase III randomized, placebo-controlled, REGAIN clinical trial, eculizumab is efficacious in acetylcholine receptor antibody (AChR-Ab)-positive refractory generalized myasthenia gravis (gMG) (Myasthenia Gravis Foundation of America—MGFA class II–IV). It has not been studied in severe myasthenic exacerbation or myasthenic crisis (MGFA V). A 73-year-old man diagnosed with myasthenia gravis AChR-Ab positivity came to our observation for symptoms of bulbar and ocular weakness and unresponsiveness or intolerability to conventional immunosuppressive therapies (prednisone and azathioprine). Due to the recurrent clinical worsening with intubation over a short-term period, the patient was treated with eculizumab. After 15 days of eculizumab treatment, we observed a significant recovery of clinical condition. We discharged the patient to an outpatient regimen, where he is continuing with maintenance doses of eculizumab and slowly tapering steroid intake. The use of eculizumab in myasthenic crises is still anecdotal. Our case aims to provide eculizumab benefit for refractory severe gMG in a practical, real-world setting beyond the criteria of the REGAIN study. Further studies are needed to evaluate the efficacy and safety of eculizumab in myasthenic crises.

## 1. Introduction

Myasthenia gravis (MG) is a chronic autoimmune complement-mediated disorder that affects neuromuscular junction triggered in about 70% of cases by pathogenic antibodies (Ab) against the acetylcholine receptor (AChR).

Activation of the complement cascade results in the formation of the membrane attack complex (MAC), the destruction of the postsynaptic end-plate, and, ultimately, the AChR(s) reduction, leading to muscle weakness and fatigue [1]. The severity of symptoms correlates with the degree and variety of muscle involvement [2,3]. Myasthenia Gravis Foundation of America (MGFA) clinical classification is used to describe MG presentations into different classes by clinical features with increasing severity of diseases (Table 1) [4]. 

About 20% of MG patients have isolated ocular involvement, while the remaining prominent part have generalized myasthenia gravis (gMG), characterized by ocular, limb, axial, and bulbar muscle affection [2]. 

AChR-Ab-positive MG is divided into early-onset (<50 years of age), late-onset (≥50 years of age), and thymoma-associated MG [3]. Early-onset MG occurs mostly in females, while late-onset MG is prevalent in males; thymoma-associated MG mainly shows late-onset and severe symptoms [2]. 

Myasthenic crisis (MGFA class V), a life-threatening condition with a mortality risk estimated at more than 10%, is characterized by rapid, severe clinical worsening, with bulbar muscle weakness and acute neuromuscular respiratory failure, and, therefore, represents the most severe complication of MG patients [2,3].

Various therapeutic options are available for MG treatment, from acetylcholinesterase inhibitors (pyridostigmine) as symptomatic drugs, to corticosteroids (oral prednisone, prednisolone) or steroid-sparing drugs (azathioprine, mycophenolate mofetil, methotrexate, tacrolimus, and cyclosporine) as long-term immunomodulatory/immunosuppressive drugs, and thymectomy in selected patients [5]. However, long-term immunomodulatory/immunosuppressive drugs need several months (6–9 months) to ensure clinical benefit with the risk of early cumulative toxicity [3]. Around 10–15% of MG patients have refractory disease, defined as nonresponsiveness or intolerable adverse events to long-term immunomodulatory/immunosuppressive treatments. Refractory myasthenic patients are a subgroup of patients with persistent disease burden, although treated with multiple drugs over the disease course, more prone to develop drug toxicity, and likely to have a worse prognosis, for whom the use of more specific therapies represents a clinical need [6].

The myasthenic crisis requires intensive cures and often invasive supportive treatment associated with short-term immunomodulatory/immunosuppressive therapies such as intravenous immunoglobulins (IVIg), plasma exchange (PEX), or immunoadsorption apheresis (IA) [3,5]. 

Among immunomodulatory/immunosuppressive treatments is eculizumab, a humanized monoclonal antibody approved by the European Medicines Agency in 2017 for treating adult patients with refractory generalized AchR-Ab-positive MG, based on results of the phase III randomized, placebo-controlled, REGAIN study [7,8].

The REGAIN study excludes individuals suffering from a myasthenic crisis at screening and who required treatment with IVIg or PEX within the 4 weeks before randomization [8].

The purpose of the case report is to present a patient who experienced treatment for refractory AChR-Ab-positive gMG with superimposed myasthenic crisis with eculizumab. 

## 2. Case Description

A 73-year-old man was referred to our clinic for a ten-day history of fluctuating head drop, swallowing disturbance, dysphonia, and asymmetric bilateral drooping eyelids with pupil-sparing. 

Brain magnetic resonance imaging was unremarkable, and computed tomography (CT) angiography was negative for acute cerebrovascular disease. At the completion of the diagnostic process, he was diagnosed with late-onset gMG AchR-Ab-positive (Myasthenia Gravis Foundation of America—MGFA IIb); chest CT was negative for thymoma. Steroids and pyridostigmine were started and titrated at efficacious and tolerated doses (pyridostigmine 60 mg thrice daily, prednisone 60 mg daily). 

Despite the therapies, he showed a gMG worsening with shortness of breath, hoarseness, and dysphagia over a few weeks (MGFA IV b) managed with noninvasive ventilation and prompt administration of IVIg (2 g/Kg over five days). After achieving improvement (MGFA I), he was dismissed with pyridostigmine and maintenance of high-dose oral steroids. 

In the following three months, he developed long-term side effects of steroid use; therefore, gradual steroid tapering was carried out while he commenced azathioprine as an immunosuppressive steroid-sparing drug. Nevertheless, azathioprine was suspended after two months of exposure for idiosyncratic cholestatic reaction, with a subsequent need for increasing oral corticosteroid therapy. 

After a short period of clinical stability, symptoms of a severe relapse of gMG by bulbar weakness and respiratory failure occurred (MGFA V- myasthenic crisis), requiring transfer to the intensive care unit for invasive ventilation. The administration of IVIg (2 g/Kg over five days) was not helpful, so seven sessions of IA and steroid titration up to 1 mg/Kg/day (prednisone 80 mg daily) were used to wean from mechanical ventilation successfully. 

The patient experienced a recurrence of the myasthenic crisis triggered by an urosepsis in two weeks, managed by IA again, imbricated with intravenous antibiotics and critical care support, including intubation. 

**Figure 1:**
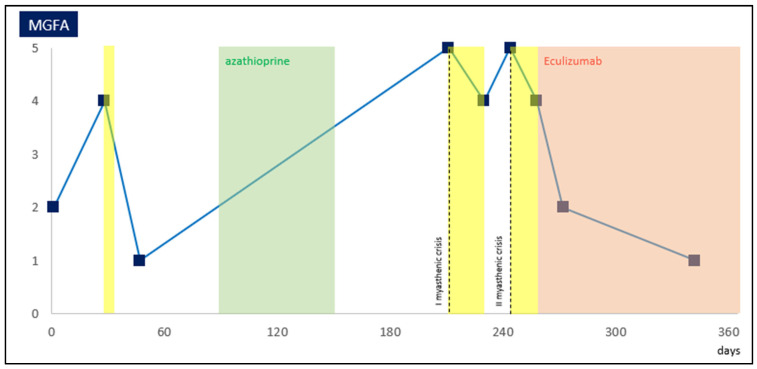
Evolution of clinical severity of gMG in our patient, assessed through MGFA. Day 0 represents clinical diagnosis, yellow translucent areas represent administration of IVIg and IA, green translucent area represents the period of azathioprine administration and suspension, and red area represents the start of eculizumab infusions, all expressed in days. Dashed lines represent a myasthenic crisis requiring ICU admission and invasive ventilation (MGFA, Myasthenia Gravis Foundation of America I–V; gMG, generalized myasthenia gravis; IVIG, intravenous immunoglobulins; IA, immunoadsorption; ICU, intensive care unit).

In light of the short-term recurrence of severe worsening and refractoriness of gMG to conventional therapies, we considered eculizumab for rapid (and long-term) improvement. After the first meningococcal vaccination, the loading dose of eculizumab (900 mg intravenously weekly for four weeks) was given while severe oropharyngeal muscle weakness remained predominant (MGFA IVb). Following the first two inductive infusions, the patient showed a significant improvement in bulbar weakness, categorized as MGFA IIb, and was discharged. He progressively reached the best clinical conditions (MGFA I) in the next few weeks, till the 12th week of eculizumab infusion, so we proceeded simultaneously with slow steroid tapering (Figure 1). Until 2 weeks after the completion of the meningococcal vaccination schedule, the patient received a course of antibiotic prophylaxis. 

After six months, while he continues to receive the maintenance doses of eculizumab therapy at a dosage of 1200 mg intravenously every two weeks, no adverse events or disease relapse have arisen, and he currently shows no signs of the disease.

## 3. Discussion

The long-term immunomodulation/immunosuppression obtained with the use of corticosteroids and steroid-sparing drugs is nonspecific to the pathological mechanism of AchR-positive MG and could be flawed for several rationales [3].

Eculizumab, as a first-approved therapy targeting complement specifically binding with high affinity to a human terminal complement protein component, has revolutionized the treatment of MG. 

It exerts its effects by inhibiting the enzymatic cleavage of complement component C5 by the C5-convertase, thereby inhibiting its partition in C5a (a potent anaphylotoxin and chemotaxin) and C5b, which recruits complement components C6, C7, C8, and C9 [9]. The resulting C5b formation blockage prevents the C5b-9 MAC formation [8,9]. However, at the same time, eculizumab preserves complement component C3 cleavage for C3a and C3b generation, which is essential for microorganism opsonization and triggers phagocytosis [9]. The complement cascade inhibition by blocking MAC formation in experimental models has been demonstrated to prevent disease onset and reverse its progression [10]. 

In addition to AchR-Ab-positive gMG, eculizumab showed efficacy in paroxysmal nocturnal hemoglobinuria (PNH), atypical hemolytic uremic syndrome (aHUS), and aquaporin-4 (AQP-4)-Ab-positive neuromyelitis optica spectrum disorder (NMOSD) [11,12,13]. Serum concentrations of free C5 < 50 μg/mL pharmacodynamically correlate with drug activity resulting in complete complement blockade in gMG, PNH, aHUS, and NMOSD [3].

In the randomized, double-blind, placebo-controlled REGAIN study (ClinicalTrials.gov, NCT01997229), eculizumab has proven efficacious in treating refractory AchR-Ab-positive gMG patients [8]. Despite the failure to reach the primary endpoint of a reduction in the Myasthenia Gravis-Activities of Daily Living Scale (MG-ADL) score, an eight-item patient-reported scale developed to assess MG symptoms and their effects on daily activities from baseline to week 26, likely due to the worst-rank analysis of covariance adopted (*p* = 0.0698), the post hoc sensitivity worst-rank analysis of covariance showed significant improvements in MG-ADL score using its reduction of at least three points in the prespecified sensitivity analysis as one of the secondary outcomes of the study. Most of the improvement was achieved by 12 weeks, even if this had already occurred in the first weeks [8,14]. Hence, although the primary endpoint of the REGAIN study was not met, the analysis of the secondary endpoints showed the potential benefit of eculizumab, as confirmed in the open-label extension (OLE) study [15].

In the 3-year REGAIN OLE study (ClinicalTrials.gov, NCT02301624), eculizumab was shown to be able to improve the status of about 90% of patients, and “minimal manifestations of the disease” were achieved in about 60% of patients; additionally, it demonstrated a steroid-sparing effect in about 50% of the enrolled patients [15].

Rituximab is another therapeutic option commonly used for long-term treatment of refractory MG and typically takes up to more than 3 months to show an impact on the disease course [16]. In the RINOMAX trial (ClinicalTrials.gov, NCT02950155), rituximab was demonstrated to achieve the status of “minimal disease manifestation” after 16 weeks of treatment [17]. It acts as a monoclonal antibody targeting the CD20 protein, leading to B cell depletion (and the synthesis of new antibodies). Although it seems to ensure a good safety profile for long-term therapy for gMG patients, with the available evidence to date, it is unattainable to corroborate the overall clinical benefit of rituximab in AChR-positive gMG patients. In a recent network meta-analysis by Saccà et al., rituximab appeared to be a treatment strategy that did not show statistically significant differences compared to placebo in reducing scores on the MG-ADL as well as the patient’s quality of life, in contrast to eculizumab [18,19]. MuSK-positive MG patients benefit more from rituximab therapy [16]. The main distinctive characteristic of antibodies against MuSK concerning anti-AChR Ab is that they do not activate the complement-cascade-mediated damage at the neuromuscular junction [20].

Instead, eculizumab provides evidence of faster and sustained efficacy. A post hoc analysis of the REGAIN study and its open-label extension study highlighted the rapid and sustained clinical benefit due to eculizumab treatment. In the placebo/eculizumab group, significant improvements were observed across all muscle group domains within a few weeks (2–4 weeks) of eculizumab treatment and were maintained for up to 2.5 years [21].

Furthermore, the fast effectiveness of eculizumab is also supported by the observation of lowered serum 50% hemolytic complement activity (CH50), which measures the total activity of the terminal complement cascade already following the first inductive dose [13,22]. These data suggest the role of eculizumab as a therapeutic option in case of acute gMG worsening, in addition to IVIg, PLEX, and IA. The imbrication with these is already possible based on what is reported in the REGAIN study, where these were indicated as rescue medications for patients experiencing clinical deterioration during treatment with eculizumab [8].

Interestingly, unfavourable courses were observed in the REGAIN OLE study during eculizumab treatment, suggesting a group of patients for whom complement inhibition cannot prevent clinical deterioration [15]. Different reasons could underlie this eventuality. A rare genetic C5 complement protein mutation responsible for inadequate eculizumab binding has been described in PNH patients [23]. In addition, previous findings also inform that intercurrent acute infections and delays in treatment could reduce the efficacy of eculizumab and the chance for a complete recovery [22,24].

Our case is similar to that described by Vinciguerra et al., of a 74-year-old male patient with AChR-Ab-positive gMG who suffered from a myasthenic crisis and was not responsive to IVIg initially but favorably evolved in a short time following the start of treatment with eculizumab [25]. This case, jointly with ours, illustrates the potential value of eculizumab as rescue therapy for obtaining rapid clinical response in myasthenic exacerbation/crisis refractory to conventional treatments, even in case of late AChR-Ab-positive MG onset.

Furthermore, in addition to what is already known from the studies, also supported by our experience in a real-world clinical setting, the early positive response rates place clinicians as being able to evaluate the satisfactoriness of the treatment if a clinical benefit is not achieved within a few months (e.g., 3–6 months), resulting in interruption of treatment to also mitigate its annual cost (approximately EUR 330,000 per year) [8,15,21].

Notwithstanding, in this newly open scenario of “tailored therapy”, the most recent 2020 updated consensus guidelines for MG treatment do not indicate eculizumab as a treatment for MGFA V (severe myasthenic exacerbations and crisis) because this clinical status was one of the REGAIN study exclusion criteria [26]. However, eculizumab efficacy can be outstanding in these several situations, as reported in several other case reports and case series, and the 2020 guidelines also stress the need for further research into the efficacy of eculizumab in severe myasthenic exacerbations and myasthenic crisis [25,26,27,28,29,30,31].

## 4. Conclusions

As an innovative selective treatment for AchR-Ab-positive gMG with a short response latency, eculizumab can achieve a positive clinical response, causing limited adverse effects. In line with the literature, our experience supports the hypothesis that eculizumab is a promising therapy for severe gMG exacerbation, including myasthenic crisis. In our opinion, the early use of eculizumab during the acute phase, even without waiting for the complete meningococcal vaccination schedule associated with intensive care and accurate antibiotic prophylaxis, has increased the probability of rapid rescue. In addition, the absence of complications and the good functional recovery confirmed over the next six months and reduced oral steroids therapy provided further evidence of eculizumab’s effectiveness and tolerability. However, randomized and controlled clinical trials are needed to support the usefulness of eculizumab in patients with refractory myasthenic crises. Further studies are also necessary to identify which MG patient subgroup responds longer to the eculizumab treatment and which complement-related biomarkers could allow us to tailor the treatment strategy best. These unmet needs are particularly relevant because of the high drug costs. 

## Figures and Tables

**Table 1:** The Myasthenia Gravis Foundation of America (MGFA) clinical classification divides MG into 5 main classes and several subclasses. The classes correlate with prognosis. Myasthenic crisis or class V, according to the MGFA classification, presents itself as an exacerbation of bulbar and respiratory weakness, leading to nasogastric intubation, endotracheal intubation, or mechanical ventilation [4].

MGFA Class	Clinical Description
**Class I**	Any ocular muscle weakness; may have weakness of eye closure. All other muscle strength is normal.
**Class II**	Mild weakness affecting muscles other than ocular muscles; may also have ocular muscle weakness of any severity. **IIa**. Predominantly affecting limb, axial muscles, or both. May also have lesser involvement of oropharyngeal muscles. **IIb**. Predominantly affecting oropharyngeal, respiratory muscles, or both. May also have lesser or equal involvement of limb, axial muscles, or both.
**Class III**	Moderate weakness affecting muscles other than ocular muscles; may also have ocular muscle weakness of any severity. **IIIa**. Predominantly affecting limb, axial muscles, or both. May also have lesser involvement of oropharyngeal muscles. **IIIb**. Predominantly affecting oropharyngeal, respiratory muscles, or both. May also have lesser or equal involvement of limb, axial muscles, or both
**Class IV**	Severe weakness affecting muscles other than ocular muscles; may also have ocular muscle weakness of any severity. **IVa**. Predominantly affecting limb, axial muscles, or both. May also have lesser involvement of oropharyngeal muscles. **IVb**. Predominantly affecting oropharyngeal, respiratory muscles, or both. May also have lesser or equal involvement of limb, axial muscles, or both.
**Class V**	Defined as intubation, with or without mechanical ventilation, except when employed during routine postoperative management (The use of a feeding tube without intubation places the patient in class IVb)

## Data Availability

The data presented in this study are available on request from the corresponding author.
